# Conversion therapy for advanced hepatocellular carcinoma following complete response to transarterial radioembolization combined with atezolizumab and bevacizumab

**DOI:** 10.2478/raon-2026-0015

**Published:** 2026-03-24

**Authors:** Peter Popovic, Ana Kalamutova, Mihajlo Djokic, Anka Cuderman, Gasper Boltezar, Blaz Trotovsek

**Affiliations:** Clinical Institute of Radiology, University Medical Centre Ljubljana, Ljubljana, Slovenia; Faculty of Medicine, University of Ljubljana, Ljubljana, Slovenia; Department of Abdominal Surgery, University Medical Centre Ljubljana, Ljubljana, Slovenia; Department of Nuclear Medicine, University Medical Centre Ljubljana, Ljubljana, Slovenia; Diagnostic Center Bled Group, Bled, Slovenia

**Keywords:** advanced hepatocellular carcinoma, transarterial radioembolization, atezolizumab, bevacizumab, complete response, conversion therapy, surgery

## Abstract

**Background:**

The current Barcelona Clinic Liver Cancer (BCLC) classification recommends systemic treatment with atezolizumab and bevacizumab as the first-line therapy for advanced hepatocellular carcinoma (HCC). Recent studies suggest that combining systemic immunotherapy with locoregional treatments, such as transarterial radioembolization (TARE), may enhance immune responses and improve overall treatment outcomes. This article presents a case series of three patients with advanced hepatocellular carcinoma who were treated with transarterial radioembolization followed by atezolizumab and bevacizumab achieving conversion to surgical resection.

**Patients and methods:**

Between June 2020 and April 2024, three patients with advanced HCC were treated with TARE followed by atezolizumab and bevacizumab. The cohort included: Patient 1: A 59-year-old female, with noncirrhotic liver, with a 12 cm tumor and a 1.5 cm satellite lesion located in the liver, with hepatic vein and inferior vena cava (IVC) tumor thrombosis (Vv3 Japanese classification) and a small lung metastasis. Patient 2: A 63-year-old male with chronic hepatitis C (CHV), presenting with a 10 cm tumor and portal vein tumor thrombosis (Vp4 Japanese classification). Patient 3: A 50-year-old male, with non-cirrhotic liver, with a 17 cm tumor with portal vein and IVC tumor thrombosis (Vp3, Vv3 Japanese classification).

**Results:**

The combined treatment approach enabled surgical resection in all three patients, each achieving a complete pathological response. Interestingly, follow-up dosimetric analysis showed that all tumors had received a subtherapeutic absorbed radiation doses.

**Conclusions:**

In selected patients, combining transarterial radioembolization with systemic immunotherapy may enable conversion to surgical resection in advanced hepatocellular carcinoma, even with subthreshold tumor radiation doses, highlighting a potential synergistic and abscopal effect between locoregional and systemic therapies.

## Introduction

Hepatocellular carcinoma (HCC) is the third leading cause of cancer-related death globally.^1^ Over 50% of newly diagnosed cases are identified at an advanced stage, meaning patients are unsuitable for curative treatments such as surgery, liver transplantation or local ablative techniques.^2^ Recent advances in systemic therapy, particularly immune checkpoint inhibitors combined with anti-angiogenic agents, have improved survival outcomes in this population.^1,3-5^

According to the Barcelona Clinic Liver Cancer (BCLC) classification, transarterial radioembolization (TARE) is currently recommended only for selected patients with very early or early-stage disease.^1^ The two principal techniques are radiation segmentectomy (RS) and radiation lobectomy (RL). RS aims for complete pathological necrosis while sparing non-tumorous liver parenchyma and is used as a curative modality in early HCC patients unsuitable for ablation, resection, or transplantation. In addition, radiation lobectomy aims to achieve local tumor control while inducing hypertrophy of the contralateral liver lobe, thereby improving hepatic reserve and enabling surgical resection as a form of downstaging.^6^

Limited objective responses to conventional chemotherapy and variable resistance to immune checkpoint inhibitors (ICIs) have prompted exploration of combined systemic and locoregional strategies in HCC, such as TARE with the aim of enhancing immune responses and improving treatment outcomes.^1,3,6,7^ Internal radiation during TARE not only impacts tumor cells by making them more visible to cytotoxic T-cells, but it also stimulates the host’s immune response by activating dendritic cells and the release of proinflammatory cytokines.^8^ Furthermore, internal radiation can enhance PD-L1 expression on tumor cells, which may increase the effectiveness of ICIs.^6,8^

Recent literature describes an “abscopal effect”, where locoregional treatments induced necrosis of tumor cells releases antigens that trigger a systemic immune response by activating T cells. This can lead to the recognition and destruction of distant tumor cells, offering promising prospects for treating undiagnosed satellite tumors.^1,6,8^

Currently, the combination of locoregional and systemic therapy for advanced HCC is not yet approved in the guidelines, although recent studies suggest that integration of immune checkpoint inhibitors with locoregional therapies like TARE represents a potentially effective disease control or conversion therapy strategy for improving outcomes in advanced HCC.^6^ Conversion therapy refers to the use of systemic or locoregional treatments to downstage initially unresectable tumors to a point where curative therapies such as surgical resection or liver transplantation become possible.

## Patients and methods

In our article, we present a cohort of three patients with BCLC stage C HCC, who received treatment with TARE followed by atezolizumab/bevacizumab and conversion to surgical resection. All cases were evaluated within a multidisciplinary tumor board (MDTB) prior to treatment initiation, as the treatment approach represents a departure from current recommended management for BCLC C HCC. Written informed consent was obtained from all patients. All procedures were conducted in accordance with the ethical standards and with the 1964 Helsinki declaration and its later amendments or comparable ethical standards. Data collection was retrospective.

### Patient 1

**FIGURE 1.**
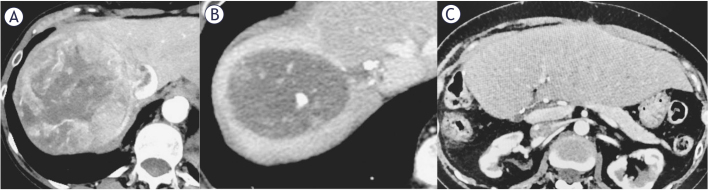
58-year-old female with a 116 mm hepatocellular carcinoma (HCC) in the right hepatic lobe, with a tumor thrombus (Japanese classification Vv3) in hepatic vein and inferior vena cava. **(A)**. Follow up CT scan after 8 months demonstrated a complete response to transarterial radioembolization (TARE) and atezolizumab/bevacizumab treatment **(B)**. Last follow-up CT scan was 7 months after surgery and showed no signs of disease progression **(C)**.

A 58-year-old female was referred to our center following an incidental finding on computed tomography (CT) revealed a hypervascular lesion in the right hepatic lobe measuring 116 mm, accompanied by a 15 mm satellite lesion and tumor thrombus (Japanese classification Vv3) in hepatic vein and inferior vena cava (IVC) (Figure 1A). Also, a small lung metastasis was present. According to the Japanese classification system for evaluating vascular invasion, in portal vein tumor thrombosis (PVTT), Vp1 indicates thrombus located beyond second order branches, Vp2 thrombus located in the second order branches, Vp3 thrombus located in the first order branches, whereas Vp4 indicates a thrombus located in the main portal vein. For hepatic vein tumor thrombosis, Vv1 denotes involvement of a peripheral hepatic vein, Vv2 a major hepatic vein, and Vv3 the inferior vena cava.^9^

Her medical history included emphysematous chronic obstructive pulmonary disease (COPD) as consequence of prolonged smoking history, arterial hypertension, and osteoporosis, with no prior abdominal surgeries. Her performance status was 0. Laboratory evaluation showed preserved synthetic liver function with serum bilirubin level of 10 μmol/L, albumin level of 50g/L and albuminbilirubin (ALBI) score of -3.59. Alpha-fetoprotein (AFP) was 7.9 kU/L.

An ultrasound-guided needle biopsy confirmed HCC in a non-cirrhotic liver, and the patient was staged as BCLC C. Considering the patient’s overall condition, the MDTB recommended TARE followed by immunotherapy as the initial treatment strategy.

A planning angiography confirmed the tumor’s vascular supply from the right hepatic artery. To minimize potential lung shunting coil embolization of a small vessel perfusing the tumor thrombus in right hepatic vein was performed. 99mTc-macroaggregated albumin (MAA), 74 MBq, was administered via the right hepatic artery. Whole body planar scintigraphy and SPECT/CT of the abdomen revealed a hepatopulmonary shunting measuring 15%, no other extrahepatic deposition and a suitable tumor uptake. The prescribed activity of ^90^Y resin microspheres (SIR-spheres; Sirtex Medical Limited) was calculated using BSA (body surface area) method, with an additional activity reduction due to lung shunt, resulting in the final activity of 1059 MBq. Radiation lobectomy was performed without complications, and the patient was discharged the following day.

At three months post-TARE, CT showed a 7% reduction in primary tumor size (from 116 mm to 108 mm), increased of IVC tumor thrombus (from 31 mm to 38 mm), stable satellite lesion, and a 7 mm metastasis in the lower right lung lobe.

4 months after TARE, atezolizumab and bevacizumab therapy was initiated. She tolerated treatment well, reporting only upper limb paraesthesia after the first cycle.

During 13 cycles over 9 months, CT scans showed a partial response (PR) after 3 cycles according to modified response evaluation criteria in solid toumors (mRE-CIST) (34% size reduction) and complete response (CR) after 11 cycles. The MDTB deemed the tumor resectable (Figure 1B) and she underwent right hemihepatectomy with lymphadenectomy and partial IVC resection. Pathohistology showed a complete tumor necrosis with no residual vital tumor, tumor thrombus in IVC without viable cells, and eight negative lymph nodes. Hospitalization was complicated by pneumonia, treated successfully with antibiotic, patient was discharged on postoperative day (POD) 12.

Follow-up every three months showed no disease progression (Figure 1C). The patient died from primary cardiac arrest 7 months post-surgery. Autopsy revealed no evidence of malignant disease. The overall survival (OS) was 20 months.

### Patient 2

**FIGURE 2.**
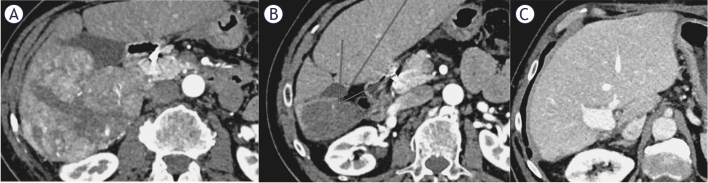
63-year-old male with a 100 mm hepatocellular carcinoma (HCC), chronic hepatitis C (CHV), without cirrhosis, with portal vein tumour thrombosis (Japanese classification Vp4) before treatment **(A)**. Patient received transarterial radioembolization (TARE) and 13 cycles of atezolizumab/bevacizumab, achieving complete response on follow-up CT scan after 14 months **(B)**. Subsequently, surgical resection was performed, with no signs of disease progression **(C)**.

A 69-year-old male presented with 3-month of right upper quadrant pain and unintentional weight loss. Ultrasound revealed a large right hepatic lobe lesion, confirmed on CT as 100 mm involving segments 5 and 6, with main portal vein tumor thrombus (Vp4). No extrahepatic disease was identified (Figure 2A).

The patient had a past history of arterial hypertension and treated chronic hepatitis C (CHV, genotype 1b), achieving sustained virological response (SVR) 6 years prior. He had no prior abdominal surgeries.

An ultrasound-guided biopsy confirmed HCC, he had Child A cirrhosis, with a score of 6 points, performance status (PS) 1, bilirubin 12 μmol/L, albumin 40g/L, ALBI score -2.69) and AFP 524 kU/L. The MDTB recommended TARE followed by immunotherapy.

Planning angiography showed tumor supply from the right hepatic artery. Coil embolization of the gastro-duodenal artery prevented reflux. 168 MBq of ^99m^Tc-MAA was injected into the right hepatic artery. Whole body planar scintigraphy and SPECT/CT of the abdomen revealed a hepatopulmonary shunting measuring 2%, no other extrahepatic deposition and a very heterogenous tumor uptake. The prescribed activity of ^90^Y resin microspheres calculated with BSA method was 1225 MBq. The procedure was followed by radiation lobectomy, with no periprocedural complications observed. Discharge was the next day.

At months, CT showed PR with reduced tumor and thrombus size. Atezolizumab/bevacizumab was started 4 months after TARE. Side effects including double vision, pruritus, cough, nightmares and hypothyroidism, managed with levothyroxine. The patient received 13 cycles of immunotherapy.

At 9 months after starting atezolizumab/bevacizumab, follow-up CT demonstrated CR according to mRECIST (Figure 2B). The MDTB deemed the HCC resectable, and a right hemihepatectomy with segmental portal vein resection and end to end reconstruction was performed. Pathohistology showed necrotic tissue with dystrophic calcification and surrounding inflammatory reaction, without residual tumor. Discharged on POD 6 with no recorded complications.

53 months follow-up revealed no disease progression (Figure 2C).

### Patient 3

A 50-year-old male was referred to our center with a one-year history of upper abdominal pain and unintentional weight loss of 10 kg. Imaging, including abdominal ultrasound and CT scans of the abdomen and thorax, showed a 170 mm lesion involving segment 6^th^, 7^th^ and partially 5^th^ and 8^th^ with posterior branch right portal vein invasion and suspected 16 mm left lobe metastasis. No extrahepatic spread was detected.

An ultrasound-guided biopsy confirmed macrotrabecular and focally pseudoglandular well-differentiated HCC in the right lobe, the left lobe lesion was negative. The patient had no significant past medical history and with PS 0, bilirubin 27 μmol/L, albumin 39 g/L, ALBI score -2.37, AFP 2.5 kU/L.

The MTDB initially deemed the tumor potentially resectable and recommended surgical exploration. Intraoperatively, the tumor was found unresectable due to macrovascular invasion and tumor thrombosis of IVC.

**FIGURE 3.**
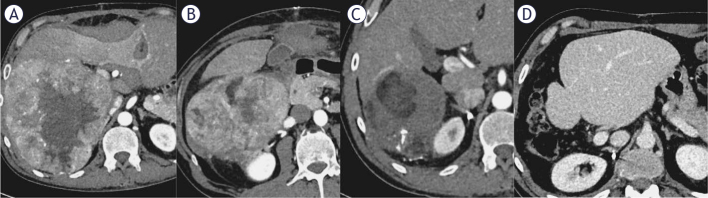
50-year-old male with 170 mm hepatocellular carcinoma (HCC), portal vein tumor thrombosis (Japanese classification Vp4), inferior vena cava (IVC) tumor thrombosis (Japanese classification Vv3) and Child A cirrhosis **(A)**. Follow-up CT scan showed a partial response 2 months after transarterial radioembolization (TARE) **(B)**, followed by 7 cycles of atezolizumab/bevacizumab, resulting in a complete response after 4 months **(C)**. A right hemihepatectomy with marginal excision of the vena cava was performed, with no disease progression upon follow up **(D)**.

Follow-up CT imaging confirmed right portal vein branch tumor thrombosis (Vp3), tumor extended into the right hepatic vein, with tumor thrombus in the IVC (Vv3). Tumor was reclassified as unresectable BCLC C stage and the patient was considered a candidate for TARE and immunotherapy (Figure 3A).

Planning angiography revealed a complex tumor perfusion. The right posterior sectoral branch supplied approximately 60% of the tumor, while the remaining supply came from three branches of the extrarenal part of the right renal artery and a dominant branch from the intrarenal part of right renal artery. 159 MBq of ^99m^Tc-MAA was injected into the right posterior sectoral branch of hepatic artery while 75 MBq of ^99m^Tc-MAA was injected into the dominant branch from the renal hilus.

Whole-body planar scintigraphy and SPECT/CT showed 12% lung shunt, with no other extrahepatic tracer deposits and heterogeneous tumor uptake.^90^Y glass microspheres (Therasphere, Boston Scientific Corp., calculated with multicompartment partition dosimetry) were delivered via hepatic and renal branches (3,3 GBq + 1,4 GBq) combined with coil embolization of two renal artery branches supplying the tumor and adjacent extrahepatic structures, as confirmed by Cone Beam CT. Additionally, drug-eluting bead transarterial chemoembolization (DEB-TACE) was performed, delivering 75 mg of doxorubicin loaded on DC Bead Lumi microspheres into a branch of the renal artery, which was estimated to supply up to 20% of the tumor. According to the predictive dosimetric calculations, the perfused parts of the tumor received 100 Gy, which is far below the minimum tumor absorbed dose threshold of 205 Gy.^10^ The injected ^90^Y glass microsphere activity could not be increased because the 12% lung shunt reached the limiting safety factor of 30 Gy lung absorbed dose. The patient was discharged the following day without complications.

One month later, patient began atezolizumab/bevacizumab. 2 months post TARE and DEB-TACE, CT showed PR according to mRECIST (Figure 3B), followed by 7 cycles of immunotherapy, resulting in CR according to mRECIST criteria with 35% reduction in size of the lesion at 4 months (Figure 3C).

MDTB deemed tumor resectable and in patient underwent right hemihepatectomy with marginal IVC excision. Pathohistology showed necrotic tissue with surrounding inflammatory reaction, with no residual tumor. Patient was discharged on POD 7 with no complications.

Thirteen months post-surgical resection, the patient showed no disease progression (Figure 3D). Patient continued with adjuvant treatment with atezolizumab/bevacizumab for 12 months after surgical resection.

## Results

In our case series, all three patients had unresectable lesions with macrovascular invasion, preserved performance status (0 or 1) and either noncirrhotic liver or Child A cirrhosis. Portal hypertension was excluded making them suitable for atezolizumab/bevacizumab treatment. One patient received TARE +DEB-TACE due to complex tumor perfusion pattern with the slow release of doxorubicin enhancing local ischemic effects.^11^ Key results are summarized in Table 1. Dosimetric analysis showed subthreshold absorbed doses in the first two patients (55 Gy and 45 Gy, respectively, instead of the expected 100 Gy threshold for resin dosimetry) due to BSA-based resin microsphere calculation.^12^ In the third patient, personalized dosimetry planning was used, but a large lung shunt limited the injected activity of ^90^Y glass microspheres, yielding an absorbed tumor dose of 100 Gy, far below the 205 Gy threshold for glass microsphere dosimetry. No periprocedural complications or treatment related toxicity events were observed after TARE. Systemic treatment was well tolerated in all three, without discontinuation.

**TABLE 1.** Summary of key results

Variable	Patient 1	Patient 2	Patient 3
**Age / Sex**	58 / Female	69 / Male	50 / Male
**Liver cirrhosis**	Non-cirrhotic liver	Child–Pugh A (6 points)	Non-cirrhotic liver
**Performance status**	0	1	0
**Baseline AFP (kU/L)**	7.9	524	2.5
**Tumor size (mm)**	116 + 15 mm satellite lesion	100	170
**Vascular invasion**	Hepatic vein + IVC (Vv3)	Main portal vein (Vp4)	Portal vein (Vp3) + hepatic vein + IVC (Vv3)
**Extrahepatic disease**	Lung metastasis (7 mm)	None	None
**TARE type**	^90^Y resin microspheres	^90^Y resin microspheres	^90^Y glass microspheres
**Additional locoregional therapy**	None	None	DEB-TACE
**Absorbed tumor dose (Gy)**	55	45	100
**Time to immunotherapy**	4 months post-TARE	4 months post-TARE	1 month post-TARE
**Immunotherapy cycles**	13	13	7 preoperatively + 12 adjuvant
**Best radiological response (mRECIST)**	CR after 8 months	CR after 14 months	CR after 4 months
**Serious adverse events following TARE + atezo/beva**	No	No	No
**Surgical procedure**	Right hemihepatectomy + partial IVC resection	Right hemihepatectomy + portal vein resection	Right hemihepatectomy + marginal IVC excision
**Pathological response**	Complete necrosis, no viable tumor	Complete necrosis, no viable tumor	Complete necrosis, no viable tumor
**Postoperative complications**	Pneumonia	None	None
**Length of stay (POD)**	12	6	7
**Follow-up outcome**	No recurrence; died of cardiac arrest	No recurrence at 53 months	No recurrence at 13 months
**Overall survival**	20 months	Ongoing (> 53 months)	Ongoing (> 13 months)

Atezo/beva = atezolizumab and bevacizumab; CR = complete response; DEB-TACE = drug-eluting bead transarterial chemoembolization; IVC = inferior vena cava; mRECIST = modified response evaluation criteria in solid toumors; POD = postoperative days; TARE = transarterial radioembolization; Vp3 = portal vein tumor thrombosis in Japanese classification; Vv3 = inferior vena cava tumor thrombosis in Japanese classification

According to mRECIST, all three patients achieved CR, making them candidates for surgical resection, with pathology confirming complete tumor necrosis.

## Discussion

The 2022 BCLC update emphasizes a personalized approach to HCC management, intergrating patient characteristics, tumor burden, vascular invasion and extrahepatic spread, with MDTB evaluation as a central component for clinical decisonmaking.^1^

According to the 2025 European Association for the Study of the Liver (EASL) guidelines, for patients with a high tumor burden, locoregional therapies, used either alone or in combination with systemic treatments, aim to reduce tumor volume, slow down disease progression, and enable tumor downstaging to potentially allow curative surgery.^13^

Successful downstaging is defined as a reduction in tumor size and number, enabling patients to meet the Milan Criteria (MC) for liver transplantation (LT) or allow surgery.^14,15^ Importantly, this status should be maintained for at least a 6 months post - treatment. In a study from Mazzaferro *et al*., patients who received definitive treatment with LT after downstaging demonstrated significantly higher 5- year overall survival (76.8% *vs*. 18.3%) and lower tumor recurrence rates compared to the control group.^14^ Complete pathological response following locoregional treatment prior to LT or surgery, has been associated with improved survival (OS) and lower recurrence (OS).^15^

Portal vein tumor thrombosis is a poor prognostic factor in advanced HCC, associated with a median survival of 2-6 months without treatment.^16^ Trials including HIMALAYA and IMbrave150 demonstrated that regimens combining dur-valumab/tremelimumab or atezolizumab/bevacizumab showed superior OS compared to sorafenib monotherapy.^4,5^ Specifically, IMbrave150 trial reported a 42% lower hazard of death with atezolizumab/bevacizumab, compared to sorafenib with a median OS of 19.2 months.

In a study by Kudo *et al*., 110 patients with initially unresectable BCLC-B HCC treated with atezolizumab/bevacizumab achieved a CR rate of only 1.8% and a PR rate of 34.5% based on mRE-CIST criteria.^17^ Meta-analyses corroborate that CR to atezolizumab/bevacizumab or durvalumab/tremelimumab in advanced hepatocellular carcinoma remains rare: 5.5% in IMbrave150 and 3.1% in HIMALAYA.^4,5,18^

Personalized TARE dosimetry has demonstrated significant benefits in converting unresectable HCC, including those with PVTT to resection candidates.^10,19^ The DOSISPHERE-01 trial compared multicompartment personalized dosimetry with standard lobar single-compartment dosimetry. In the personalized group, 96% of patients received tumor absorbed dose > 205 Gy, versus 42% in the standard group, yielding an objective response rate (ORR) of 71% *vs*. 36%. Median OS was 26.6 months in personalized group, with downstaged patients (35% *vs*. 4%) achieving 53% 5-year OS.^10,19^

In a larger retrospective study, Park *et al*. evaluated 213 treatment-naive HCC patients with PVTT: median OS was 27.5 months in the TARE group versus 8.6 months in atezolizumab/bevacizumab group, with survival benefit greatest in segmental or lobar PVTT (Vp1-3).^20^

To date, only two studies comparing TARE + atezolizumab/bevacizumab and one case report have been published. Villalobos *et al*. reported 19 patients with advanced, unresectable HCC receiving TARE within 3 months of systemic immunotherapy. Among these, 10 patients were treated with the combination of atezolizumab and bevacizumab, while 9 received nivolumab. ORR at 1 and 6 months was 58%, CR 16% overall, median OS 12.9 months.^2^ Yu *et al*. described 10 patients with intermediate or advanced HCC treated with sequential or concurrent TARE + atezolizumab/bevacizumab, showing 100% tumor control in TARE-treated lesions, with progression-free survival (PFS) rates at 6 and 12 months of 78.8% and 66.7% and OS of 90% at 6 months and 77.1% at 12 months.^21^ Park *et al*. presented a case report of infiltrative HCC with PVTT treated with concurrent TARE plus atezolizumab/bevacizumab, demonstrated marked tumor reduction and excellent tolerability.^22^

A large retrospective analysis of 1,664 BCLC C patient compared TARE + immunotherapy (n = 142) versus immunotherapy alone (n = 1522), showing median OS 19.8 *vs*. 9.5 months, despite heterogeneous immunotherapy regimens.^23^

Independently, both TARE and atezolizumab/bevacizumab outperform sorafenib in OS, with a lower adverse event - related therapy discontinuation.^4,24^ For TARE a clear dose-effect relationship exists, and personalized dosimetry allows higher tumor absorbed doses while maintaining safe non-tumoral liver and lung exposure.^12,25^

Our study further supports evidence from the existing literature suggesting that the combination of locoregional therapy and immunotherapy represents a promising strategy for achieving curative-intent treatment in advanced HCC. The outcomes observed in our case series are consistent with previously reported successful tumor downstaging in patients with initially unresectable HCC and macrovascular invasion, while our patients demonstrated higher recurrence-free and overall survival (Table 2). Interestingly, in our series complete pathologic necrosis occurred despite subthreshold tumor radiation doses, suggesting that lower radiation may enhance tumor antigen release and potentiate immune checkpoint blockade efficacy.^26,27,28^ This challenges the traditional focus on achieving maximal tumor absorbed doses for TARE in combination therapy.

**TABLE 2.** Comparison of literature reported transarterial radioembolization (TARE) + immunotherapy treatment

Study	Study design	No. of patients	HCC stage	Locoregional therapy	Immunotherapy regimen	CRR	Median OS
**Villalobos et al.^2^**	Retrospective	19	Advanced unresectable HCC (84% BCLC C)	TARE	Atezo/Bev (n = 10); nivolumab (n = 9)	16%	12.9 months
**Yu et al.^21^**	Retrospective	10	60% BCLC C, 40% BCLC B	TARE	Atezo/Bev	75%	90% (6 months), 77.1% (12 months)
**Yeo et al.^23^**	Retrospective	142	BCLC C	TARE	Mixed ICIs	Not reported	19.8 months
**Presen study**	Case series	3	BCLC C	TARE (± DEBTACE)	Atezo/Bev	**100%**	Ongoing; no recurrence at 13–53 months

Atezo/Bev = atezolizumab and bevacizumab; BCLC = Barcelona clinic liver cancer classification; DEB-TACE = transarterial chemoembolization; HCC = hepatocellular carcinoma; CRR = complete response rate; OS = overall survival; TARE = transarterial radioembolization

This study is limited by the small number of patients included and its retrospective, single-center design, which precludes definitive conclusions regarding efficacy and generalizability. Although promising, TARE with atezolizumab/bevacizumab requires further prospective validation to determine optimal patient selection, sequencing, and toxicity management. Our findings support the emerging paradigm that combining locoregional therapy with immunotherapy may enable downwards stage migration in BCLC C HCC.

## Conclusions

Our findings suggest that TARE combined with atezolizumab/bevacizumab may be an effective strategy to achieve tumor downstaging and enable conversion therapy in patients with advanced hepatocellular carcinoma, even when tumor radiation doses are below threshold.
